# Population Genetic Characteristics of Siberian Roe Deer in the Cold Temperate Forest Ecosystem of the Greater Khingan Mountains, Northeast China

**DOI:** 10.3390/biology13110935

**Published:** 2024-11-16

**Authors:** Xinxin Liu, Yang Hong, Jinhao Guo, Ning Zhang, Shaochun Zhou, Lu Jin, Xiaoqian Ma, Ziao Yuan, Hairong Du, Minghai Zhang, Jialong Wang

**Affiliations:** 1 College of Wildlife and Protected Area, Northeast Forestry University, Harbin 150040, China; liuxinxin2017@126.com (X.L.); hy1624@126.com (Y.H.); guojinhao19960206@126.com (J.G.); 18846797329@163.com (N.Z.); jinluft@163.com (L.J.); dhr9012@163.com (H.D.); 2 Wildlife Research Institute of Heilongjiang Province, Harbin 150010, China; zhoushaochun2003@163.com (S.Z.); maxiaoqian_79@163.com (X.M.); 3 College of Life Science and Technology, Harbin Normal University, Harbin 150080, China; yiwuanemail@126.com; 4 Institute of Applied Microbiology, Heilongjiang Academy of Sciences, Harbin 150010, China

**Keywords:** Siberian roe deer (*Capreolus pygargus*), genetic diversity, genetic differentiation, genetic structure, population dynamics

## Abstract

This study focuses on the Siberian roe deer population in the Greater Khingan Mountains, Northeast China. The cold temperate forest ecosystem in this area is distinctive. The Siberian roe deer is a crucial ecological indicator species, and its living conditions hold significant importance for ecological balance. From the winter of 2019 to 2022, 269 fecal samples of Siberian roe deer were collected from four protected areas in the northern part of the Greater Khingan Mountains, Heilongjiang Province. These samples were comprehensively analyzed using mitochondrial DNA and microsatellite markers, combined with conservation genetics evaluation methods. The results revealed that 244 individuals were identified in the fecal samples. The results of a Cyt b genetic analysis of the samples indicated that the haplotype and nucleotide diversity were 88.1% and 20.3%, respectively. The evaluation of 14 pairs of microsatellite loci showed that the average number of alleles was 11.2, and the average expected and observed heterozygosity were 0.672 and 0.506, respectively. Therefore, the overall genetic diversity level is high, but some populations of Siberian roe deer are at risk. AMOVA analysis and STRUCTURE Bayesian clustering confirmed the existence of obvious genetic differentiation among the populations. Historical studies have shown that the HZ and SH populations underwent the earliest diffusion events, and the BJC and SL populations also exhibited related signs (HZ: Huzhong Nature Reserve in the Greater Khingan Mountains; SH: Shuanghe National Nature Reserve in Heilongjiang Province; BJC: Heilongjiang Beijicun National Nature Reserve; SL: Songling District in Heilongjiang Province). Mismatch distribution and neutral tests indicated no expansion events or bottleneck effects in the population, and the inbreeding coefficient was positive, suggesting the possibility of inbreeding. The development potential of the population in the future varies among the various local populations. This study supports the biodiversity of Siberian roe deer at the genetic level to save the population and provides important scientific basis and reference for the protection and management of Siberian roe deer.

## Simple Summary

This study analyzes fecal samples from Siberian roe deer in the Greater Khingan Range of Heilongjiang Province, using mitochondrial DNA and microsatellite markers to explore the population’s genetic characteristics. The findings indicate a high level of genetic diversity within the Siberian roe deer population, alongside notable genetic differentiation among groups. Furthermore, this research offers vital insights for the conservation and management of Siberian roe deer and provides valuable data for the protection of local Northeast tigers (*Pantheratigris altaica*).

## 1. Introduction

The Greater Khingan Range in Heilongjiang Province is China’s only cold-temperate forest ecosystem. As a vital ecological barrier in Northeast China, it plays a central role in regulating climate, conserving water resources, and preventing soil erosion [1,2,3,4]. This region, with its unique geographical features and rich biodiversity, is home to numerous rare and endangered species, including the Siberian tiger (*Pantheratigris altaica*) and red deer (*Cervus elaphus*), making it a critical area for biodiversity conservation in China [5,6]. Maintaining the health of the Greater Khingan Range ecosystem is essential for protecting these flagship species and ensuring the long-term conservation of the entire regional biodiversity [7]. Furthermore, the forest vegetation in this region plays a pivotal role in absorbing carbon dioxide through photosynthesis, regulating the local climate, and acting as an important carbon sink, thereby contributing to global efforts in mitigating climate change [8,9]. Therefore, the ecological security and sustainable development of Northeast China are closely tied to the health and stability of the Greater Khingan Range [10,11].

The Siberian roe deer (*Capreolus pygargus*) is listed as one of China’s three national endemic animals. This species plays an important ecological role in the boreal forest ecosystem of the Greater Khingan Mountains and is vital to the ecological balance and stability of the region. The gnawing behavior of the Siberian roe deer has a significant impact on the distribution and structure of vegetation, and its selective feeding on different plant species results in the regulation of vegetation growth and reproduction. By controlling the growth and distribution of vegetation, the Siberian roe deer shapes the vegetation structure and diversity pattern of the entire ecosystem to a certain extent [12]. In the Greater Khingan Mountains, the Siberian roe deer plays the role of population regulator; its numbers and activities have a direct impact on the distribution and abundance of other animal and plant populations, thereby helping to maintain the ecological balance among various biological populations [13]. Maintaining Siberian roe deer populations helps to preserve the stability of the Greater Khingan Mountains ecosystem. This deer species coexists with other herbivores in the region, and their unique feeding habits and ecological niches jointly create an intricate ecological network, thereby promoting the diversity and stability of the Greater Khingan Mountains ecosystem [14]. The Siberian roe deer was once widely distributed in mountainous forest areas in Northeast China, but its population size and distribution range have declined [15,16,17]. Studies have shown that human activities are the main reason for the decline in the number and distribution range of Siberian roe deer [18,19,20,21,22].

The genetic structure of populations is a key factor in their ability to adapt to environmental changes, especially important for species facing anthropogenic pressures. Previous genetic studies of Siberian roe deer in various regions have shown that populations are increasingly isolated due to habitat fragmentation, leading to reduced gene flow and heightened risks of inbreeding. In regions such as the Russian Far East, where Siberian roe deer populations also reside, genetic studies have demonstrated significant population differentiation and low genetic diversity, which are associated with historical bottlenecks and human-induced fragmentation [23]. Similar trends have been observed in other ungulate species within cold temperate ecosystems. For example, studies on red deer populations in the Scottish Highlands and northern European regions have shown a decline in genetic diversity linked to isolation and restricted gene flow [24,25]. These findings highlight the importance of maintaining or re-establishing gene flow between fragmented populations to preserve genetic diversity and the long-term viability of species.

Recent advances in high-throughput genome sequencing technologies have revolutionized conservation genetics, providing more accurate and comprehensive insights into the genetic characteristics of endangered species [25,26]. In particular, the study of population genetics has proven invaluable for designing evidence-based conservation strategies. For example, genome-wide sequencing efforts have been pivotal in assessing the genetic health of endangered species, such as the European bison (*Bison bonasus*) and wild boar (*Sus scrofa*), where targeted conservation actions, including translocation and habitat restoration, have been implemented based on genetic findings [27,28]. In light of these advances, this study aims to contribute to the conservation efforts for Siberian roe deer in the Greater Khingan Mountains by investigating the population genetic characteristics of the species in this unique cold-temperate forest ecosystem. By examining these genetic features, we aim to provide essential scientific insights that can guide future conservation actions to restore and protect Siberian roe deer populations in the region. Understanding the genetic diversity, population structure, and gene flow of Siberian roe deer in this context is crucial not only for their recovery but also for the broader ecological restoration of the area, including the preservation of predator–prey dynamics involving the Siberian tiger [19,24].

## 2. Materials and Methods

### 2.1. Study Area

**Figure 1:**
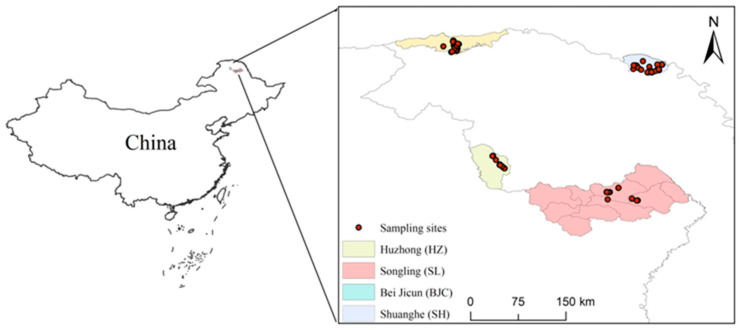
The sampling map for Siberian roe deer from different locations: Bei Jicun (BJC), Huzhong (HZ), Shuanghe (SH), and Songling (SL) regions.

Heilongjiang Beijicun National Nature Reserve is located in the northern part of Yilehuli Mountain in the Greater Khingan Mountains forest area (121°40′00″–123°16′00″ E, 53°11′30″–53°33′03″ N, Figure 1), with a total area of 137,553 km^2^. The terrain in the reserve is relatively gentle and belongs to the inland mountain area. It is mainly composed of medium-sized mountains, low mountain hills, and plain hills along the river. The overall terrain is high in the south and low in the north. Shuanghe National Nature Reserve in Heilongjiang Province is located in the northeast of the Greater Khingan Mountains in China [22,29] (124°52′48″–125°32′03″ E, 52°54′25″–53°12′08″ N, Figure 1), with a total area of 888.49 km^2^. The terrain of the reserve is flat, of which about 90% of the total area is distributed in the gentle slope area below 15° [22]. Huzhong Nature Reserve in the Greater Khingan Mountains (122°42′14″–123°18′05″ E, 51°17′42″–51°56′31″ N, Figure 1) is sandwiched between the main range of the Greater Khingan Mountains and Yilehuli Mountain. It is 63 km long from north to south and 32 km wide from east to west, with a total area of 167,213 hm^2^. The area has steep terrain, densely covered with towering peaks and a complex geological structure, with an overall terrain pattern of high in the west and low in the east. Songling District in Heilongjiang Province is located in the northern part of the Greater Khingan Mountains. It is a medium–low mountain area with complex and changeable terrain and undulating mountains (123°29′23″–125°50′07″ E, 50°09′16″–51°23′48″ N, Figure 1). The winters in the Greater Khingan Mountains are long, dry, and cold, with average annual temperatures of −4 to −4.3 °C; the lowest extreme temperatures are −45.8 to −53 °C [22]. The frost period lasts from early September to late May of the following year, the freezing period lasts up to seven months, the snow accumulation period averages 165 to 175 days, and the thickness of the permafrost layer is about 2.5 to 3.0 m [30,31]. In some low-lying swampy areas, island-like permafrost can even be observed [22].

### 2.2. Sample Collection DNA

From November 2019 to January 2022, random plots were set up at four research sites. During the plot survey, footprints or lying traces of Siberian roe deer were found around them, and fecal samples were found and collected in disposable sterile collection bags. During the sample collection process, the samples were temporarily placed outdoors in a low-temperature environment and protected from light to ensure the integrity and stability of fecal DNA. When all sample collection work was completed, the samples were placed in a −80 °C ultra-low-temperature refrigerator in the laboratory for long-term storage. A total of 269 samples were collected (Table 1).

### 2.3. Extraction and Species Identification

In this study, we extracted and amplified DNA from the feces of Siberian roe deer. The laboratory operating procedures were strictly followed during the experiment to prevent contamination of foreign DNA in the PCR products. First, fecal DNA was extracted using the QIAamp Fast DNA Stool Mini Kit (Qiagen, Hilden, Germany) according to the manufacturer’s instructions. Then, the mtDNA Cyt b primers L14724 (5′-CGAGATCTGAAAAACCATCGTTG-3′) and H15149 (5′-AAACTGCAGCCCCTCAGAATGATATTTGTCCTCA-3′), universal for deer, were selected for PCR amplification of fecal DNA [32,33,34]. The amplification system is shown in Table S1. The reaction conditions were as follows: 95 °C pre-denaturation for 3 min; 95 °C denaturation for 30 s, 53 °C annealing for 10 s, and 72 °C extension for 30 s, for a total of 35 cycles; and finally, 72 °C extension for another 5 min, followed by storage at 4 °C. At the same time, each amplification was performed with laboratory-stored muscle DNA as a positive control (derived from naturally dead individuals found in the wild), and a negative control without DNA was added to monitor contamination. PCR products were checked using 1% agarose gel electrophoresis, and positive products were sent to Shanghai Sangon Biotech Co., Ltd. (Shanghai, China) for purification and bidirectional sequencing. DNAStar Lasergene version 17.2.1 was used to splice, align, and correct the forward and reverse sequences, which were compared with the NCBI GenBank reference database to identify the Siberian roe deer species [35]. If the sample was not identified as Siberian roe deer, it was discarded and no further analysis was performed.

### 2.4. Individual Identification

Based on published studies on ungulates, 14 pairs of microsatellite primers (T507, BM848, IDVGA8, BM1706, T156, BM757, BMC1009, ILSTS008, T108, CSSM43, Roe06, T530, CSSM41, and MCM131) were used for individual identification [36,37,38,39,40]. The 5′ end of the upstream primer for each microsatellite locus was fluorescently labeled, and the downstream primer was unlabeled (Table S2). Reaction conditions were the same as for species identification, except for the return temperature (Table S2). The polymerase chain reaction was performed several times at each locus using a multitube method to obtain reliable genotypes [41]. Genotypes with an estimated reliability probability of 95% were accepted using RelioType software [42]. Each locus was amplified at least four times using polymerase chain reaction analysis. Negative controls were set up, and PCR products were checked using 1% agarose gel electrophoresis. Genotypes were determined using an ABI 3730XL sequencer and the GeneMapper version 5.0 (Applied Biosystems Inc., Waltham, MA, USA). The Excel microsatellite toolkit was used to search for matching genotypes in the data [43]. The identification principles included the following: the genotypes at all microsatellite loci were exactly the same; there was only one microsatellite locus with allele differences [22,44,45].

### 2.5. Genetic Diversity Analysis

The analysis of mtDNA sequences and microsatellite data can reveal genetic variation and evolutionary relationships between species. This article introduces the method of using ClustalX 2.1 software to arrange Cyt b sequences and DnaSP 5.10 software to calculate various indicators [46,47]. First, MEGA 11 software was used to align the Cyt b sequences of different individuals [48]. The number of Cyt b sequence variation sites (*S*) and the number of haplotypes (*H*) were calculated using DnaSP 5.10 software, and the haplotype diversity index (*H_d_*) and nucleotide diversity index (*P_i_*) of the four populations were calculated. The four populations were taken as a whole, and the *H_d_* and *P_i_* of the Greater Khingan Mountains population of Siberian roe deer were calculated [22,47,49].

For the microsatellite data, Gimlet version 1.3.3 was used to evaluate the individual identification probability (*PID*) of 14 microsatellite loci [50]. Microchecker version 2.2.0.3 was used to detect whether there were invalid alleles or allele losses at microsatellite loci to ensure data quality [51]. Genepop version 4.0 was used to measure whether the population and each locus deviated from Hardy–Weinberg equilibrium (*H_WE_*) [52], and the linkage disequilibrium (LD) between each locus was checked. For the LD and HWE tests, *p*-values were generated using the Markov chain method, and significance was corrected using the Bonferroni method [53]. GenAlEx version 6.5 was used to convert the allele data and calculate the number of alleles (*N_a_*), the number of effective alleles (*N_e_*), the expected heterozygosity (*H_o_*), and the expected heterozygosity (*H_e_*) [54]. Parameters such as allele richness (*AR*) and inbreeding coefficient (*F_is_*) were calculated through FSTAT version 2.9.3.2, and the data of each microsatellite locus were calculated through the software PIC-CALC to obtain the polymorphic information content value (*PIC*) [55].

### 2.6. Genetic Differentiation Analysis

The genetic distances of four Siberian roe deer populations were calculated using MEGA11 software based on the Cyt b gene sequences [48]. In the study of the microsatellite data, the software Genetix 4.05 was used to calculate the genetic differentiation index (*F_st_*) and gene flow (*N_m_*) [56], and a significance test repeated 1000 times was performed to determine the reliability of the results. In addition to *F_st_*, genetic differentiation among populations was assessed by analysis of molecular variation (AMOVA). Different from *F_st_*, AMOVA uses genetic distance to evaluate differences between populations. GenAlEx 6.0 was used to conduct AMOVA analysis [54], and the significance of the results was verified via simulation. The software STRUCTURE version 2.3.4 was used to analyze the population’s genetic structure [57]. The population number (K value) was set from 1 to 10, and 20 repeated operations were performed for each K value. The calculation results were uploaded to Structure Harvester version 0.6.94 to analyze the posterior probability LnPr(X|K) and the ∆K values to determine the most suitable K value [58]. The software CLUMPP version 1.1.2 was used to average the 20 repeated results [59], and the grouping results of each individual were visually presented. Excel 2003 was used to complete this process. Through the above methods, the genetic differentiation among populations was comprehensively assessed, and the population structure was effectively identified.

### 2.7. Population Dynamics Analysis

By using the rapid expansion model in DnaSP 5.10 software, base pair frequency differences between mitochondrial DNA haplotypes were analyzed to understand the dynamic history of the population [38]. When conducting mismatch distribution analysis, it should be noted that insufficient sample size may lead to conflicting or erroneous results. Therefore, this study first conducted a neutrality test analysis on the populations in the four regions, namely Tajima’s D and Fu’s Fs tests, to detect evidence of population expansion [60,61]. When Tajima’s D is significantly greater than 0, bottleneck effects and balancing selection can be inferred; when Tajima’s D is significantly less than 0, group size amplification and directional selection can be inferred [62,63].

For the detection of bottleneck effects, this study selected the Wilcoxon one-tailed test as the main analysis method, using the two-way mutation model (TPM) and stepwise mutation model (SMM) in Bottleneck1.2 software to perform microsatellite marker analysis with 1000 repetitions [22,64]. At the same time, this study used microsatellites as neutral molecular markers, combined with Genepop 4.0, Myriads 1.1, and GenAlEx 6.0 software, to conduct Hardy–Weinberg equilibrium tests and effective population size assessments on different local populations to reveal the relationship between genetic characteristics and inbreeding effects within isolated small populations [52,53,54]. GenAlEx 6.0 was used to calculate the observed heterozygosity and expected heterozygosity of each population [54], and the inbreeding coefficient (*F_is_*) was further obtained to evaluate the impact of inbreeding on the population [65]. In addition, the LDNe 1.31 software was used to estimate the effective population size of each local population and compare it with the actual population size to explore the proportion of effective population size in isolated small populations [22,66]. Under conditions of small sample sizes, the software LDNe 1.31 is an effective tool for estimating the effective population size (*N_e_*), and its calculations provide a reliable reflection of the number of genetically effective individuals in the population [66].

## 3. Results

### 3.1. Species Identification and Individual Identification

**Figure 2:**
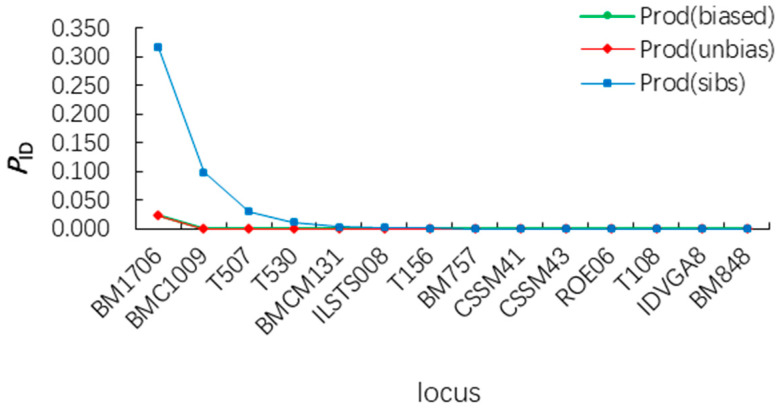
Individual genotypic similarity probability curves of 14 microsatellite loci order. Note: Prod(unbias): This term refers to the “parental unbiased” genotype reconstruction method. Prod(sibs): This term refers to the “siblings-based” genotype reconstruction method.

A total of 269 fecal samples were collected in the study area, and 263 DNA samples were successfully extracted, with a sample utilization rate of 97.78%. The Cyt b gene (490 bp) was successfully amplified from 263 samples. After Blast comparison, 255 samples were finally identified as Siberian roe deer. Gimlet analysis showed that the total Prod(unbias) of 14 microsatellite loci was 2.33 × 10^−2^. Even in the case of full siblings, the misjudgment probability Prod(sibs) was only 0.388%. When most polymorphic loci failed to amplify, Prod(sibs) rose to 0.463%, which was still less than 1% (Figure 2). Individual identification results showed that a total of 244 independent Siberian roe deer individuals were identified from 255 Siberian roe fecal samples and used for subsequent analysis (Table S3).

### 3.2. Genetic Diversity Analysis

By splicing and screening 244 Cyt b sequences that were 490 bp in length, this study found a total of 267 variant sites, including 41 single variant sites and 291 parsimony information sites. Among these variant sites, there are 74 missing sites. The overall G + C content is 35.5%, indicating that the genome of this species has certain base composition characteristics. A total of 53 different haplotypes were detected. Haplotype analysis was conducted on the four populations of BJC, SL, HZ, and SH, and 30, 16, 21, and 26 haplotypes were found, respectively. The overall haplotype diversity index (*H_d_*) and nucleotide diversity index (*P_i_*) of the Siberian roe deer populations at the four locations were calculated, and the results showed that *H_d_* was 0.881 ± 0.017 and *P_i_* was 0.203 ± 0.134. Among them, the *H_d_* of the BJC population was the highest, at 0.913 ± 0.022, while the SL population showed a lower haplotype diversity of only 0.734 ± 0.004 (Table 2). In contrast, the nucleotide diversity index (*P_i_*) of the BJC population was 0.089 ± 0.068, while the SH population had the lowest, at only 0.011 ± 0.040.

Microsatellite genetic diversity showed that the number of alleles (*N_a_*) in the four populations ranged from 8.5 to 13.7, with an average of 11.2, and the number of effective alleles (*N_e_*) ranged from 4.2 to 5.6, with an average of 5.0 (Table 3). Compared with the actual observed number of alleles, the effective number of alleles at each site was significantly lower, and the difference was extremely significant (*p* < 0.01), which may imply the risk of allele loss (Table 3). The allelic richness (*AR*) range of the four populations ranged from 9.629 to 11.735, and the overall level was relatively high. The polymorphic information content (*PIC*) of individual microsatellite loci ranged from 0.469 to 0.912. Overall, the entire population significantly deviated from the Hardy–Weinberg equilibrium at 14 sites, and the inbreeding coefficient (*F_is_*) values were all positive, indicating that there was a certain degree of heterozygosity deficiency in the population.

### 3.3. Genetic Differentiation Analysis

Genetic distance between local populations (Table 4), the microsatellite data results show that there is significant genetic differentiation among Siberian roe deer populations in different regions (*p* < 0.001) (Table 5). Overall, this species shows a very significant level of genetic differentiation (*F_st_* = 0.16285, *p* < 0.001). Gene flow (*N_m_*) between populations is relatively low, with Nm between the BJC and SH populations being 0.91011, and *N_m_* between all other population pairs being greater than 1 (Table 6). The analysis of the molecular differences within populations indicates that internal differences account for 84% of the total variation, while differences between populations only account for 16% of the total variation (Table S4).

**Figure 3:**
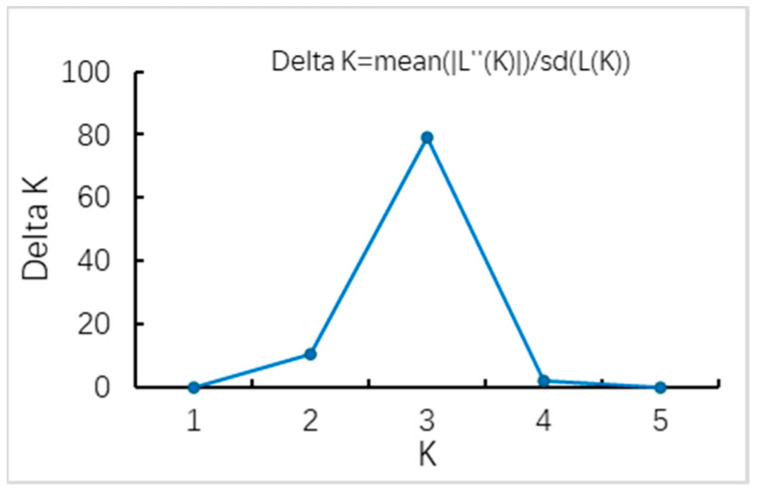
Trend of ΔK from the clustering results of the microsatellite data.

**Figure 4:**
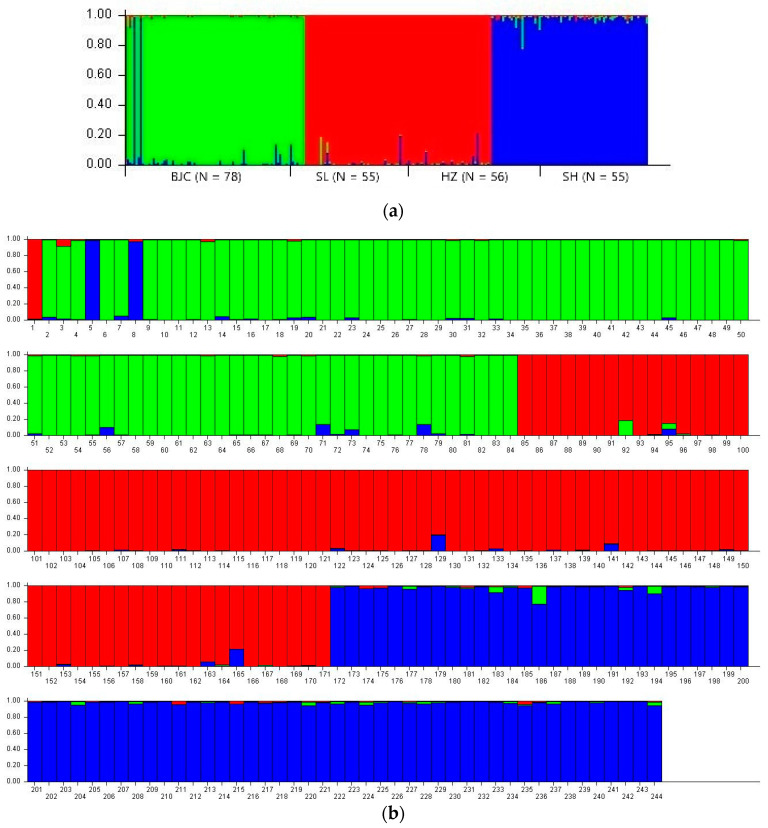
Bayesian genetic clustering analysis of the microsatellite data (K = 3). Note: Each colored vertical bar represents an individual. The proportion of vertical bars with different colors illustrates the probability that individuals are assigned to various groups. (**a**) The letters at the bottom indicate the sample collection place. (**b**) The numbers indicate the number of individuals. BJC: 1–78, SL: 79–133, HZ: 134–189, SH: 190–244.

Bayesian clustering shows that when K = 3, the mean value of LnPr(X|K) reaches the maximum, and the fluctuation range between repeated experiments is small, which indicates that the stability of the model is enhanced under the assumption of K = 3 [17,22]. The genetic grouping of 244 individuals showed significant diversity. It is worth noting that there is obvious genetic differentiation between the SH population and other populations, while genetic exchanges between other populations are more frequent and the genetic similarity is higher. In particular, the genetic similarity between the SL and HZ populations is extremely significant, which indicates that there is a close genetic connection between the two populations, which plays a key role in maintaining the genetic diversity and adaptability of the overall population (Figure 3 and Figure 4).

### 3.4. Population Dynamics Analysis

**Figure 5:**
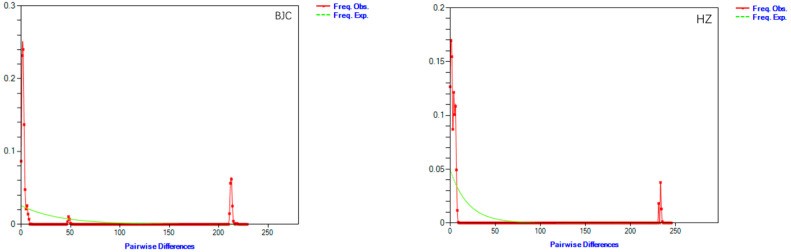

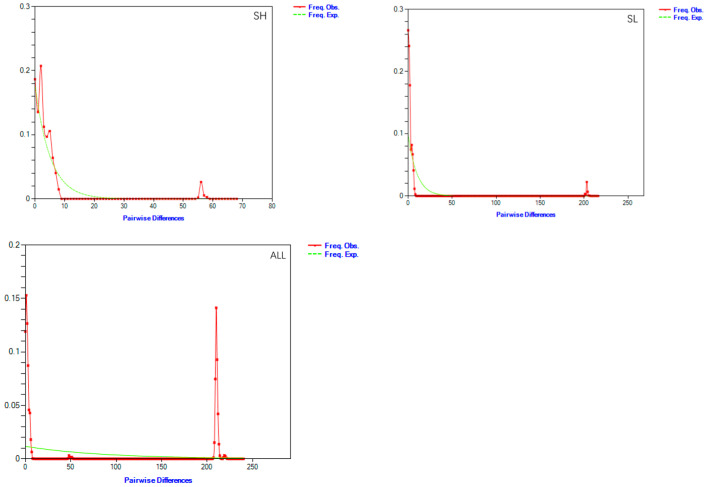
Mismatch distribution map of Siberian roe deer population. Note: Y axis–frequency corresponding to each pairwise difference.

We used T (Myr) to estimate the population expansion time, where T = π/2 (ut) [61]. It can be inferred from the dispersal time that populations in the four areas experienced dispersion during the Quaternary Pleistocene; at the same time, the BJC population and the Pine Ridge population experienced dispersion during the last glacial period of the Quaternary. Combining the mismatch distribution map and the neutrality test results, it can be seen from the population diffusion time that the HZ (T = 0.121358 Myr) and SH (T = 0.109400 Myr) populations have the earliest diffusion times, followed by the BJC (T = 0.062921 Myr) and SL (T = 0.041852 Myr) populations. By estimating the expansion times of the populations, we found that the HZ and SH populations had relatively long dispersal times, while the BJC and SL populations mainly experienced the dispersal process of the Quaternary Pleistocene and the Last Glacial Age. A mismatch distribution map was constructed based on the observed and expected frequencies of base pairs between Cyt b gene haplotypes, showing that the population did not expand (Table 7, Figure 5). The HZ and SH populations showed significant negative values (*p* < 0.05), while the SL population showed extremely significant negative values (*p* < 0.01); the Tajima’s D of the overall population was −2.06599, showing a borderline significant negative value (*p* = 0.04875 < 0.05). Calculating the Cyt b gene Fu’s Fs value, BJC, SL, and HZ were all positive, while SH showed an insignificant negative value (*p* > 0.1). A combination of mismatch distribution analysis and neutrality testing showed that no recent historical expansion events occurred in the four populations.

The detection results of population bottleneck effects based on the microsatellite data show that the Siberian roe deer population in the Greater Khingan Mountains has not experienced a recent bottleneck effect. In the TPM model, four populations showed significant heterozygosity deficiency *(p* < 0.01), suggesting that these populations did not suffer from recent bottlenecks. In both the SMM and TPM models [22], the heterozygosity excess index results showed no significant difference (*p* > 0.05), and the allele frequency distribution did not deviate significantly from the typical “L”-shaped distribution. There is no evidence of near-term bottleneck effects in other populations. In the SMM model, the detection results of insufficient heterozygosity in the four populations were extremely significant (*p* < 0.01), which was consistent with previous research results (Table 8). Taking into account the results of the mitochondrial DNA neutrality test, the Wilcoxon test of the microsatellite data, as well as the SMM (stepwise mutation model) and TPM (two-phase model), it can be inferred that the Siberian roe deer population has not experienced recent expansion events in the evolutionary process [22].

The effective population size was studied and compared, and the results showed that the modern effective population size ratio of the overall population of Siberian roe deer was 79.18%. However, there were obvious differences between populations in different regions. The SH population had the highest effective population size ratio, reaching 91.27%, indicating a high genetic diversity. The BJC and HZ populations had population size ratios of 87.95% and 86.96%, respectively, also indicating high levels of genetic diversity. Among the 55 Siberian roe deer in the SL population, only 25.7 were identified as effective populations, suggesting that this population may face a greater genetic risk (Table 9).

## 4. Discussion

In-depth studies on the distribution and population dynamics of Siberian roe deer (*Capreolus pygargus*) within the cold temperate forest ecosystem of the Greater Khingan Mountains provide valuable insights into the species’ demographic history. The earliest population surveys date back to the 1950s, with a report by the Institute of Zoology, Chinese Academy of Sciences, between 1953 and 1957, highlighting a decline in population due to excessive hunting [22,67]. Continued monitoring, including genetic studies, is essential for understanding the long-term survival and adaptation of the species to environmental changes. The current study extends our understanding by assessing both mitochondrial and microsatellite genetic diversity, providing a comprehensive view of population health and genetic resilience.

Genetic diversity is fundamental to species’ ability to adapt to environmental changes. In this study, the microsatellite data show relatively high genetic diversity across Siberian roe deer populations in the Greater Khingan Mountains. The average number of microsatellite alleles per population ranged from 8.5 to 13.7, with an overall average of 11.2, a value higher than that reported for the Muling population between 2018 and 2021 (10.222 ± 1.092) [49]. The observed high levels of genetic diversity suggest that these populations are well-equipped to adapt to environmental pressures. However, the presence of significant inbreeding, indicated by positive Fis values and reduced heterozygosity in all populations, reveals a paradox. While Siberian roe deer populations show high genetic diversity at certain loci, the genetic structure shows a deficiency in heterozygosity, characteristic of inbreeding [22,44]. This suggests the potential risk of further genetic deterioration if such trends continue, indicating that local populations, despite their diversity, may be vulnerable to inbreeding depression. This paradox highlights the critical importance of maintaining genetic variability to avoid the long-term loss of adaptive potential.

The *F_is_* value, a measure of heterozygote deficiency within a population, is particularly relevant in this study. Positive *F_is_* values indicate a greater degree of inbreeding, where individuals are more likely to mate with close relatives, leading to a reduction in genetic diversity. In this study, all populations exhibited positive *F_is_* values, which is statistically significant and suggests widespread inbreeding. The magnitude of these values underscores the potential genetic risks associated with such trends, including the loss of allelic diversity, reduced fitness, and an increased susceptibility to genetic disorders. This finding aligns with the broader ecological literature, where similar inbreeding patterns have been observed in other ungulate populations inhabiting fragmented or isolated ecosystems. For example, studies on moose (*Alces alces cameloides*) and Soay sheep (*Ovis aries*) have shown that high levels of inbreeding can result in reduced reproductive success and genetic health [68,69]. Given the isolation of Siberian roe deer populations in the Greater Khingan Mountains, these populations may face similar risks, emphasizing the need for careful genetic management.

Analysis of genetic differentiation among populations reveals restricted gene flow across the study area. The microsatellite data show significant genetic differentiation (*F_st_* = 0.16285, *p* < 0.001) among the Siberian roe deer populations, with generally low gene flow (*N_m_*), particularly between the BJC and SH populations. Gene flow among other populations is somewhat higher, indicating that the topography and landscape features of the Greater Khingan Mountains may act as barriers to genetic exchange. These findings are consistent with previous studies showing that geographic isolation and environmental factors can limit gene flow in forested ecosystems [22]. While some populations, such as SL and HZ, show more frequent genetic exchange, the overall connectivity remains relatively low, suggesting that the populations are in a fragmented state, which may threaten their long-term genetic health. Bayesian clustering analysis supports this conclusion, revealing distinct genetic groups among the four populations, with the SH population being clearly differentiated from others. Notably, the BJC and SL populations show closer genetic affinities, likely due to overlapping habitats or shared ecological corridors. The closer genetic relationship between the SL and HZ populations may play a vital role in preserving genetic diversity across the entire Siberian roe deer population. However, the restricted gene flow between more distantly related populations, such as those in the SH region, further underscores the need for targeted conservation strategies that promote connectivity and prevent further isolation.

Population expansion analyses based on the mitochondrial Cyt b gene indicate that Siberian roe deer populations in the Greater Khingan Mountains likely experienced dispersal events during the Quaternary Pleistocene, with some populations, such as those in HZ and SH, dispersing earlier than those in BJC and SL. Despite these historical movements, the current mismatch distribution and neutrality tests show no evidence of recent population expansions, suggesting that the population is relatively stable or possibly experiencing demographic stasis. These findings are consistent with other studies reporting no significant recent expansion in Siberian roe deer populations in other regions [16,19,22].

Further, the microsatellite analysis on population bottleneck effects suggests no significant recent population declines. Although observed heterozygosity was lower than expected, indicating some degree of inbreeding, the absence of significant bottleneck effects (as indicated by the TPM and SMM models) suggests that the populations have maintained relatively stable sizes [70]. These findings highlight the importance of maintaining stable population sizes and avoiding future bottlenecks through effective management [71].

The effective population size (*N_e_*_)_ is a key indicator of a population’s long-term genetic health and adaptive capacity. In this study, the overall Ne ratio for Siberian roe deer (*Capreolus pygargus*) was estimated at 79.18%, suggesting a relatively stable genetic structure. However, notable variation was observed among local populations. The SH population, with an Ne ratio of 91.27%, shows high genetic resilience, while the SL population, at 25.7%, faces significant risks of genetic erosion and inbreeding [72,73]. These differences underscore the need for tailored conservation strategies. Smaller populations like SL may require genetic supplementation or habitat corridors to enhance gene flow, whereas larger, more genetically diverse populations like SH are better equipped to withstand environmental changes [74]. While no clear genetic bottlenecks or declines in diversity were found, Ne estimation is subject to limitations, such as sample size and data quality. Smaller sample sizes may lead to underestimation of *N_e_*, and errors in data can distort genetic structure [73]. Furthermore, the assumptions underlying Ne models—such as constant population size and random mating—may not always hold true in natural populations, especially when demographic fluctuations or non-random mating occur [74]. Future studies should address these limitations and employ complementary genomic approaches for more robust Ne estimates to inform conservation efforts [72].

The G + C content of 35.5% in the Siberian roe deer’s Cyt b gene sequence reflects its mitochondrial DNA base composition [19,20]. While a lower G+C content can indicate genetic stability and reduced mutation rates, it may also limit adaptive capacity and reduce mitochondrial diversity, which could have negative consequences for population health, particularly in fragmented populations [75]. Therefore, monitoring genetic diversity and enhancing gene flow remain critical for the species’ long-term viability [75].

The findings of this study have broader implications for ungulate population dynamics within the cold temperate forest ecosystem of the Greater Khingan Mountains. The interaction between various ungulate species and their competition for resources plays a significant role in shaping community structures and influencing ecosystem health [76]. Maintaining healthy Siberian roe deer populations is essential not only for their survival but also for the overall balance of the ecosystem, as they play a vital role in vegetation dynamics and nutrient cycling [19,76]. Furthermore, the results underscore the importance of Siberian roe deer conservation for protecting the Amur tiger, a top predator in this ecosystem [77]. Healthy ungulate populations support predator-prey dynamics and contribute to ecological integrity, which is crucial for the survival of apex predators [77]. Therefore, the implementation of conservation strategies aimed at enhancing genetic diversity and promoting habitat connectivity will contribute to the resilience and sustainability of both Siberian roe deer and the broader ecosystem in the Greater Khingan Mountains. These strategies may also inadvertently support the Amur tiger population, highlighting the interconnectedness of species within this ecosystem [78].

## 5. Conclusions

This study provides a comprehensive understanding of the genetic characteristics of Siberian roe deer populations in the Greater Khingan Mountains, a key region within China’s cold temperate forest ecosystem. The results demonstrate that while high genetic diversity exists in most of the populations, significant risks remain for their long-term survival due to limited gene flow and genetic differentiation, especially in the SH population. These findings underscore the importance of maintaining genetic health to prevent the adverse effects of inbreeding and genetic drift.

To mitigate these risks and promote the long-term viability of the Siberian roe deer, several strategies must be considered. In addition to enhancing external gene flow through habitat corridors and translocation programs, efforts should focus on in situ conservation measures that can directly improve genetic diversity. These may include promoting habitat heterogeneity to create diverse ecological niches, fostering population growth in key areas, and implementing genetic monitoring to identify at-risk populations. Furthermore, ex situ conservation programs, such as genetic resource banks or controlled breeding programs, could play a crucial role in bolstering the genetic pool of the species, particularly for smaller, isolated populations.

Looking ahead, future research should focus on long-term genetic monitoring to track changes in allele frequencies, heterozygosity, and inbreeding rates across different populations. Additionally, further studies on the ecological interactions between Siberian roe deer and other species in the Greater Khingan Mountains could provide valuable insights into how these populations can be supported within the broader ecosystem. Research on the effects of environmental changes, such as climate change or habitat destruction, on genetic diversity will also be critical in shaping effective conservation strategies.

In conclusion, while the Siberian roe deer populations in the Greater Khingan Mountains exhibit notable genetic diversity, addressing the risks of fragmentation and limited gene flow is essential. Combining both genetic management and landscape-level conservation efforts, alongside continued research into the ecological dynamics and genetic health of these populations, will be vital for ensuring the long-term persistence of Siberian roe deer in this unique ecosystem.

## Figures and Tables

**Table 1:** Fecal sample information of Siberian roe deer.

Collection Site	Area Code	Fecal Sample/Individual Number
Shuanghe Nature Reserve	SH	57/55
Beijicun Nature Reserve	BJC	91/78
Huzhong Nature Reserve	HZ	61/56
Songling District	SL	60/55

**Table 2:** Genetic diversity analysis of Siberian roe deer population based on the Cyt b data.

Population	BJC	SL	HZ	SH	All
Individual numbers	78	55	56	55	244
Number of variation sites (*S*)	243	208	240	71	267
(G + C) content (%)	35.7	35.5	36.7	36.8	35.5
Number of haplotypes (*H*)	30	16	21	26	53
Haplotype diversity (*H_d_*)	0.913	0.734	0.873	0.879	0.881
Nucleotide diversity (*P_i_*)	0.089	0.020	0.043	0.011	0.203

**Table 3:** Genetic diversity parameters of four local populations based on 14 microsatellite loci.

Population	N	*N_a_*	*N_e_*	*AR*	*P_IC_*	*H_o_*	*H_e_*	*F_is_*	*P_HW_*
BJC	78	13.7	5.6	11.735	0.705	0.648	0.732	0.1159	0.0000 *
SL	55	11.7	4.9	10.908	0.707	0.503	0.729	0.3096	0.0000 *
HZ	56	10.9	5.4	9.857	0.637	0.450	0.656	0.3141	0.0000 *
SH	55	8.5	4.2	9.629	0.545	0.424	0.570	0.2568	0.0000 *
All	244	11.2	5.0	14.777	0.774	0.506	0.672	0.2468	0.0000 *

Note: *P_HW_*—probability of Hardy–Weinberg equilibrium test; *—significant *P_HW_* value (*p* < 0.001).

**Table 4:** Genetic distances between local populations based on the Cyt b data.

Population	BJC	SL	HZ	SH
BJC	—			
SL	0.0000873	—		
HZ	0.0005526	0.0003822	—	
SH	0.4242587	0.4606505	0.4586260	—

**Table 5:** *F_st_* between local populations based on microsatellites.

Population	BJC	SL	HZ	SH
BJC	—			
SL	0.18201	—		
HZ	0.18744	0.04777	—	
SH	0.21550	0.17751	0.09026	—

**Table 6:** The average number of migrants per generation among four populations of Siberian roe deer based on the microsatellite data (*N_m_*).

Population	BJC	SL	HZ	SH
BJC	—			
SL	1.12352	—		
HZ	1.08378	4.98290	—	
SH	0.91011	1.15839	2.51978	—

**Table 7:** Mismatch distribution analysis and neutral test results of four Siberian roe deer populations based on the Cyt b data.

Population	Index	BJC	SL	HZ	SH	ALL
Cyt b	π	1.72656	1.14844	3.33008	3.00195	2.30176
	(CI = 95%)	2.11328	4.90039	4.45508	7.08008	4.63721
	θ0	0.07207	0.00703	0.01230	0.00703	0.02461
	θ1	99,999.00000	99,999.00000	99,999.00000	99,999.00000	99,999.00000
	T (Myr)	0.06292	0.04185	0.12136	0.10940	0.0326
	SSD	0.01892	0.00131	0.00860	0.00477	0.00840
	(*p*-value)	0.05000	1.00000	0.35000	0.75000	0.53750
	*R*	0.04722	0.01306	0.02955	0.01714	0.02674
	(*p*-value)	0.45000	1.00000	0.75000	1.00000	0.80000
	Tajima’s D	−0.80069	−2.86308	−2.27819	−2.32201	−2.06599
	(*p*-value)	0.19300	0.00000	0.00100	0.00100	0.04875
	Fu’s Fs	16.33067	0.99140	6.27297	−3.80414	4.94773
	(*p*-value)	0.99700	0.71100	0.94500	0.11000	0.69075

**Table 8:** Wilcoxon test of population genetic bottleneck effect based on the microsatellite data.

Population		BJC	SL	HZ	SH
SMM	Heterozygosity excess	0.99979	0.99942	0.99939	0.99915
	Heterozygosity deficiency	0.00031 **	0.00076 **	0.00085 **	0.00122 **
TPM	Heterozygosity excess	0.998322	0.99786	0.97607	0.96802
	Heterozygosity deficiency	0.00269 *	0.00269 *	0.02869 *	0.03857 *
	Mode shift	Normal L-shaped distribution	Normal L-shaped distribution	Normal L-shaped distribution	Normal L-shaped distribution

Note: *—*p* < 0.05, **—*p* < 0.01.

**Table 9:** Effective population size and proportion of four populations of Siberian roe deer based on the microsatellite data.

Population	BJC	SL	HZ	SH	All
Population size	78	55	56	55	244
Effective population size (*N_e_*) (Pcrit ≥ 0.05)	68.6	25.7	48.7	50.2	193.2
Ratio	87.95	46.73	86.96	91.27	79.18

## Data Availability

The Cyt b and microsatellite data of Siberian roe deer can be obtained by contacting the author (liuxinxin2017@126.com).

## Supplementary Materials

The following supporting information can be downloaded at: https://www.mdpi.com/article/10.3390/biology13110935/s1, Table S1: PCR amplification system of Siberian roe for mtDNA and microsatellite; Table S2: Details of fourteen microsatellite loci used for the study; Table S3: Sample information of Siberian roe deer; Table S4: Molecular variation analysis table of Cyt b gene in four populations of Siberian roe deer.
