# Supplementary material for: Population Genetic Characteristics of Siberian Roe Deer in the Cold Temperate Forest Ecosystem of the Greater Khingan Mountains, Northeast China
Source: Biology (Basel). 2024 Nov 16;13(11):935. doi: 10.3390/biology13110935 (PMC11591672; doi:10.3390/biology13110935)
Supplement: Supplementary file 1 [file biology-13-00935-s001.zip › Table S1.PCR amplification system of Siberian roe for mtDNA and microsatellite.pdf]

**Table S1.** PCR amplification system of Siberian roe for mtDNA and microsatellite.

| Reagent | Cyt b (μl) | Microsatellite (μl) |
|---|---|---|
| 2×Rapid Taq Master Mix (China, Vazyme) | 10.0 | 10.0 |
| Forward Primer (10 μMol /L) | 0.8 | 0.8 |
| Reverse Primer (10 μMol /L) | 0.8 | 0.8 |
| DNA (25-50 ng/μL) | 2.0 | 6.0 |
| ddH2O | 6.4 | 2.4 |
| Total | 20.0 | 20.0 |
