# Supplementary material for: Population Genetic Characteristics of Siberian Roe Deer in the Cold Temperate Forest Ecosystem of the Greater Khingan Mountains, Northeast China
Source: Biology (Basel). 2024 Nov 16;13(11):935. doi: 10.3390/biology13110935 (PMC11591672; doi:10.3390/biology13110935)
Supplement: Supplementary file 1 [file biology-13-00935-s001.zip › Table S2. Details of fourteen microsatellite loci used for the study.pdf]

Table S2. Details of fourteen microsatellite loci used for the study

Locus	Primer sequence (5'-3')	Allele Length (bp)	Annealing temperature (°C)
BM848[1]	F：TGGTTGGAAGGAAAACTTGG	356~368	55-58
	R：CCCTCTGCTCCTCAAGACAC		
BM1706[1]	F：ACAGGACGGTTTCTCCTTATG	238~250	55
	R：CTTGCAGTTTCCCATACAAGG		
BMC1009[1]	F：GCACCAGCAGAGAGGACATT	276~286	55-58
	R：ACCGGCTATTGTCCATCTTG		
T530[1]	F：GTCCTCACAGCAGCTCTATG	252~296	55
	R：GCATTCTTTAGAACTCCAACTG		
T507[1]	F：AGGCAGATGCTTCACCATC	140~188	58
	R：TGTGGAGCACCTCACACAT		
IDVGA8[2]	F：CTCTTGGGGGCGTGTTGTCT	209~225	55
	R：TAGCAGAAAGCACAGGAGTC		
T156[2]	F：TCTTCCTGACCTGTGTCTTG	134–234	55
	R：GATGAATACCCAGTCTTGTCTG		
ILSTS008[2]	F：GAATCATGGATTTTCTGGGG	178~188	55-58
	R：TAGCAGTGAGTGAGGTTGGC		
MCM131[2]	F：ATTCACAAAGCCGCGCTTG	82~112	55
	R：ATCAAGCTCCCCTCTTCGGT		
T108[3]	F：CATGTGGAGATAGGTAGACAGA	131~179	55
	R：CCATTCTGAGTAGCTGATTCA		
Roe06[3]	F：GTTCCTAGCCCAGTGCTC	91–109	55–58
	R：TGCAGACCTGGCAGAC		
CSSM43[3]	F：AAAACTCTGGGAACTTGAAAACTA	120–124	58
	R：GTTACAAATTTAAGAGACAGAGTT		
CSSM41[3]	F：AATTTCAAAGAACCGTTACACAGC	238–244	58
	R：AAGGGACTTGCAGGGACTAAAACA		
BM757[3]	F：TGGAAACAATGTAAACCTGGG	172–204	58
	R：TTGAGCCACCAAGGAACC		

[1]Fam, [2]Hex, [3]Rox
