# Supplementary material for: Population Genetic Characteristics of Siberian Roe Deer in the Cold Temperate Forest Ecosystem of the Greater Khingan Mountains, Northeast China
Source: Biology (Basel). 2024 Nov 16;13(11):935. doi: 10.3390/biology13110935 (PMC11591672; doi:10.3390/biology13110935)
Supplement: Supplementary file 1 [file biology-13-00935-s001.zip › Table S3.Sample information of Siberian roe deer.pdf]

Table S3. Sample information of Siberian roe deer.

Collection site	BJC	SL	HZ	SH
Collected sample numbers	91	60	61	57
Species identification samples	88	58	60	57
Fecal sample utilization rate	97.78%	96.67%	96.67%	100%
Successful genotyping samples	81	58	60	56
Identified Siberian roe deer individuals	78	55	56	55
Genotyping utilization rate	96.00%	94.80%	94.80%	94.80%
Cyt b sequence numbers	78	55	56	55
