# Supplementary material for: Population Genetic Characteristics of Siberian Roe Deer in the Cold Temperate Forest Ecosystem of the Greater Khingan Mountains, Northeast China
Source: Biology (Basel). 2024 Nov 16;13(11):935. doi: 10.3390/biology13110935 (PMC11591672; doi:10.3390/biology13110935)
Supplement: Supplementary file 1 [file biology-13-00935-s001.zip › Table S4. Molecular variation Analysis Table of Cyt b Gene in four populations of Siberian Roe Deer.pdf]

**Table S4.** Molecular variation Analysis Table of Cyt b Gene in four populations of Siberian Roe Deer.

| Source of variation | Among populations | Within populations | Total | Fixation index |
|---|---|---|---|---|
| df | 3 | 240 | 243 | |
| Sum of squares | 7961.799 | 2273.808 | 10235.607 | $F_{st}$=0.82193, |
| Variance components | 43.72981 Va | 9.47420 Vb | 1.5802 | P<0.01 |
| Percentage of variation | 0.8219 | 0.1781 | 1 | |
